# Targeting G9a decreases escalated alcohol drinking in male mice in a model of combined stress and chronic alcohol exposure

**DOI:** 10.1111/acer.70142

**Published:** 2025-09-09

**Authors:** Marcelo F. Lopez, Paulina Misztak, Howard C. Becker, Christopher W. Cowan, Ethan M. Anderson

**Affiliations:** ^1^ Department of Neuroscience Medical University of South Carolina Charleston South Carolina USA; ^2^ Department of Psychiatry and Behavioral Sciences, Charleston Alcohol Research Center Medical University of South Carolina Charleston South Carolina USA; ^3^ Department of Comparative Biomedical Sciences Louisiana State University Baton Rouge Louisiana USA; ^4^ Ralph H. Johnson Department of Veterans Affairs Medical Center Charleston South Carolina USA; ^5^ NeuroEpigenix, LLC Charleston South Carolina USA

**Keywords:** accumbens, dependence, epigenetic, kappa‐opioid receptor, therapeutics

## Abstract

**Background:**

Alcohol use disorder (AUD) is a pervasive problem in society afflicting millions of people worldwide. One reason for the prevalence of AUD is that heavy alcohol drinking can produce alcohol dependence. In addition, alcohol dependence dysregulates the body's stress systems to increase alcohol drinking. Therefore, targeting dependence‐ and/or stress‐related alcohol drinking clinically could reduce heavy drinking in patients with AUD. One key mechanism thought to contribute to behaviors associated with AUD is long‐lasting epigenetic alterations of gene expression. We recently showed that the epigenetic regulatory enzyme G9a (also known as euchromatic histone‐lysine N‐methyltransferase 2 or EHMT2) is downregulated in the nucleus accumbens (NAc) in mice by alcohol dependence produced by the chronic intermittent alcohol (CIE) model. Also, we showed that either viral‐mediated NAc G9a knockdown or a systemically administered G9a inhibitor reduced stress‐potentiated alcohol drinking by the kappa‐opioid agonist U50,488.

**Methods:**

Here, we tested whether NAc G9a knockdown reduces escalated alcohol drinking in a mouse model of combined dependence plus forced swim stress (CIE + FSS) in male mice. We also tested for changes in sucrose drinking, sucrose preference, and water consumption as controls. In addition, we tested whether systemic administration of a G9a inhibitor, UNC0642, could reduce alcohol drinking in the CIE + FSS model.

**Results:**

We found that either NAc G9a knockdown or repeated systemic UNC0642 administration reduced escalated ethanol drinking following CIE + FSS, but without altering control levels of ethanol drinking or sucrose drinking or water drinking in male mice.

**Conclusions:**

These preclinical data suggest that reducing NAc G9a levels, or suppressing its enzymatic activity, can effectively reduce potentiated alcohol drinking produced by stress and/or alcohol dependence. These data suggest that G9a inhibition holds promise as a potential therapeutic for individuals who suffer from AUD.

## Graphical abstract

Targeting dependence‐ and/or stress‐escalated alcohol drinking could help patients with alcohol use disorder (AUD). We found that reducing the activity of the epigenetic regulatory enzyme G9a (aka euchromatic histone‐lysine N‐methyltransferase 2 or EHMT2) either by a viral knockdown in the nucleus accumbens (NAc) or by repeated systemic administration of a G9a inhibitor reduces escalated ethanol drinking in a model of combined stress plus dependence. This suggests that G9a inhibition holds promise as a potential therapeutic for individuals who suffer from AUD.

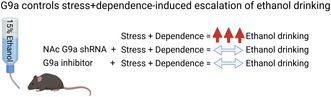

## INTRODUCTION

Alcohol use disorder (AUD) is a pervasive problem in society, afflicting millions of people worldwide (Grant et al., 2015; Mokdad et al., 2004; Sacks et al., 2015; Substance Abuse and Mental Health Services Administration, 2019). Chronic heavy alcohol drinking can result in alcohol dependence, characterized by a host of neuroadaptations and dysregulation of stress systems that serve to perpetuate harmful levels of alcohol consumption (Koob & Volkow, 2016). Therefore, targeting dependence‐ and/or stress‐related alcohol drinking clinically could be an effective strategy to reduce heavy drinking in individuals with AUD. Recent evidence indicates that epigenetic mechanisms play a key role in contributing to behaviors associated with AUD (Cruise et al., 2023; Palmisano & Pandey, 2017). Epigenetic mechanisms involve changes in nuclear chromatin structure that can alter gene expression programs, and these epigenetic changes can be very long‐lasting. Indeed, the persistent, chronic relapsing nature of AUD could be related to long‐lasting changes in gene expression and chromatin conformation in the brain. Thus, these epigenetic changes are a potential mechanism supporting the enduring brain function changes that underlie AUD.

Several epigenetic enzymes, including histone methyltransferases, are regulated by acute or chronic exposure to drugs and can influence the development of alcohol and other substance use disorders (SUD) (Anderson et al., 2023; Anderson, Penrod, et al., 2018; Anderson & Taniguchi, 2022). In some cases, alcohol/drug‐induced changes in epigenetic enzyme levels or activity are compensatory and thus constrain AUD/SUD‐related behaviors (Anderson et al., 2023, Anderson, Penrod, et al., 2018, Anderson & Taniguchi, 2022). However, in other cases, alcohol/drug‐induced changes facilitate the development or support the maintenance of AUD/SUD‐related behaviors (Anderson, Penrod, et al., 2018). One example of the former is G9a (also known as euchromatic histone‐lysine N‐methyltransferase 2 or EHMT2). G9a is a histone methyltransferase that catalyzes dimethylation on lysine 9 of histone H3 (H3K9me2) (Anderson, Penrod, et al., 2018, Covington 3rd et al., 2011, Maze et al., 2010). H3K9me2 is typically associated with condensed chromatin and repression of target gene expression, and G9a is a major regulator of this histone mark in nucleus accumbens (NAc) neurons (Anderson, Penrod, et al., 2018). Interestingly, both cocaine and opioids regulate G9a expression in the NAc (Maze et al., 2010; Sun et al., 2012). Overexpressing G9a protein levels in the NAc increases the motivation to self‐administer cocaine as measured by progressive‐ratio breakpoints, while reducing G9a expression via short hairpin RNA (shRNA) knockdown decreases this motivation. These bidirectional effects produced by manipulating G9a expression levels in the NAc were also observed in stress‐induced reinstatement of cocaine seeking—a model of stress‐induced relapse in rodents (Anderson et al., 2019; Anderson, Larson, et al., 2018). In addition, G9a expression levels in the NAc have bidirectional effects on anxiety‐like behaviors (Anderson et al., 2019, Anderson, Larson, et al., 2018).

We recently showed that G9a expression is downregulated in the nucleus accumbens (NAc) following chronic intermittent ethanol (CIE) exposure (Anderson et al., 2021), a model that produces escalated alcohol drinking in mice (Becker & Lopez, 2004; Griffin 3rd et al., 2009; Lopez et al., 2017; Lopez & Becker, 2005). Also, we showed that either viral‐mediated NAc G9a knockdown or a systemically administered G9a inhibitor reduces the ability of a pharmacological stressor (the kappa‐opioid receptor (KOR) agonist, U50,488) to increase alcohol drinking (Anderson et al., 2021). Combined, these data suggest that reducing G9a activity could be an effective strategy for treating AUD and stress‐related excessive alcohol drinking especially.

Here we tested whether G9a knockdown in the NAc reduces alcohol drinking in a mouse model of stress‐enhanced alcohol consumption involving CIE exposure combined with repeated forced swim stress exposure (CIE + FSS Drinking model) (Anderson et al., 2016b; Haun et al., 2022; Lopez et al., 2020). We also tested for changes in sucrose drinking, sucrose preference, and water consumption as controls. In addition, we tested whether chronic systemic administration of the G9a inhibitor UNC0642 could reduce stress‐enhanced alcohol drinking in the CIE + FSS model. While we detected no effects on sucrose or water drinking, our results indicate that both G9a knockdown in the NAc and repeated UNC0642 treatment reduce stress‐enhanced alcohol consumption in the CIE + FSS drinking model, but without altering moderate, baseline levels of alcohol drinking in nondependent mice. Interestingly, the G9a inhibitor UNC0642 reduced dependence‐induced escalation of alcohol drinking produced by CIE, whereas NAc G9a knockdown did not. These data suggest that reducing NAc G9a levels or G9a activity can effectively reduce potentiated alcohol drinking produced by dependence plus stress, supporting G9a inhibition as a viable candidate therapeutic for people suffering from AUD.

## MATERIALS AND METHODS

### Animals

Adult male C57BL/6J mice at least 8 weeks old (Jackson Laboratory, Bar Harbor, ME) were single‐housed in a climate‐controlled environment (21°C) on a 12‐h modified reverse light/dark cycle. Animals were habituated to the housing environment for at least 7 days prior to use in experiments, with food (Diet #2918; Harland Teklad, Madison, WI) and water available ad libitum. All behavioral experiments were performed during the dark cycle as described below. All experiments were approved by the MUSC or LSU Institutional Animal Care and Use Committee (IACUC) in facilities accredited by the American Association for the Accreditation of Laboratory Animal Care (AAALAC). All procedures were conducted in accordance with the guidelines established by the National Institutes of Health and the National Research Council.

### Viral vector stereotaxic surgery

As in our previous publications, G9a levels were reduced using an adeno‐associated vector serotype 2 containing a prevalidated short hairpin RNA (AAV‐shG9a, UNC vector core, titer: 1.2 × 10^12^); this virus has previously been shown to reduce G9a mRNA and G9a protein levels in the NAc, and we used the same AAV‐shG9a viral prep used in previously published studies (Anderson et al., 2019, 2021). A control virus (AAV‐shCTRL) was used with no known homology in mice. Both viruses expressed GFP under a CMV promoter. C57BL/6J male mice underwent isoflurane‐anesthetized survival surgery to microinject AAV‐shG9a or AAV‐shCTRL bilaterally into the NAc (AP: +1.6, DV: −4.4 ML: +1.5, 10° angle) and all mice in the study were allowed at least 21 days of recovery before any additional experimentation began to allow for peak AAV vector expression. Carprofen or ketoprofen (5 mg/kg, daily for 1–3 days as needed) was used for postsurgical pain relief. For all viral studies, brains were fixed with 4% paraformaldehyde and sliced. GFP fluorescence was used to verify NAc‐targeted expression under blinded conditions. Only mice with bilateral NAc GFP expression were included in the final analysis.

### CIE + FSS drinking model

In general, mice were given limited access (1‐h/day) to a single bottle of alcohol (15% (v/v)) in their home cage for 5 days/week for 4 weeks to establish stable alcohol intake. Alcohol access was presented at 3 h after the onset of the dark cycle. Mice were weighed weekly. After establishing stable baseline intake, mice are then typically divided into groups (balanced for baseline alcohol intake): CTL (Air‐noFSS), FSS‐alone (Air‐FSS), CIE‐alone (CIE‐noFSS), CIE + FSS. Mice that received chronic intermittent ethanol (CIE) exposure (CIE‐alone and CIE + FSS groups) were exposed to alcohol vapor (16‐h/day for 4 days) in inhalation chambers. Immediately before placement in inhalation chambers, CIE‐exposed mice received an intraperitoneal injection of alcohol (1.6 g/kg) and the alcohol dehydrogenase inhibitor pyrazole (1 mmol/kg), administered together in a volume of 0.02 mL/g body weight. Mice in the CTL and FSS‐alone groups received an injection of pyrazole only. Alcohol concentration in the inhalation chambers was monitored daily, and air flow was adjusted to maintain exposure within a range that yielded stable blood–alcohol levels within the targeted range (180–250 mg/dL) throughout each weekly exposure cycle. Blood samples were collected from the retro‐orbital sinus immediately upon removal from the inhalation chambers and assayed using an Analox Instrument analyzer (Lunenburg, MA, USA). CTL and FSS‐alone groups were similarly handled, but were maintained in control (air‐only) inhalation chambers. After an 80‐h abstinence period, test drinking sessions resumed for 5 consecutive days as during baseline. Mice in the FSS‐alone and CIE + FSS groups experienced brief (10‐min) FSS exposure 4 h prior to each of the test drinking sessions. Water was replaced before each subject, and it was maintained at 23–25°C. Upon completion of each FSS session, mice recovered in their home cage on a heating pad for 5 min and then promptly returned to the colony room. The remaining nonstressed mice (CTL and CIE‐alone groups) were left in their home cage undisturbed. Alcohol consumption during baseline and test drinking sessions was determined by weighing drinking bottles before and after the sessions.

### Evaluating the effects of viral G9a knockdown in NAc on ethanol drinking in the CIE + FSS

**FIGURE 1:**
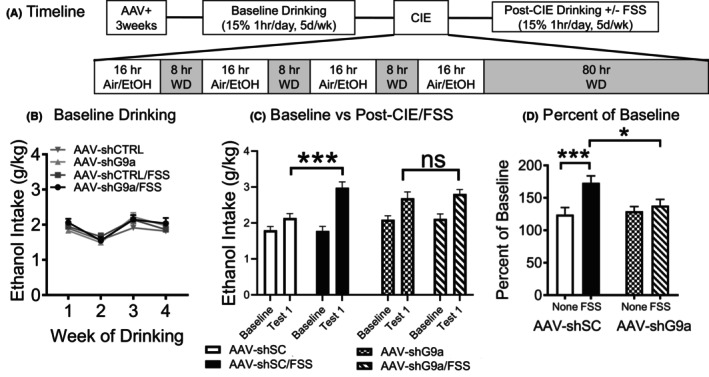
Accumbal G9a knockdown blocks stress‐induced escalation of alcohol drinking in a combined model of stress plus dependence. (A) Timeline of experiment. (B) Average ethanol drinking at baseline. (C) Average ethanol drinking following the first CIE‐FSS cycle as compared to the final week of baseline alcohol drinking. (D) A percent of baseline graph comparing ethanol drinking following the first CIE‐FSS cycle as compared to the final week of baseline alcohol drinking. Data are expressed as mean ± SEM. *p < 0.05; and ***p < 0.001. *N* = 12–13 male mice per group.

Three weeks after the NAc injection surgery, baseline drinking sessions commenced. In this experiment, after the fourth week of baseline drinking, all mice were exposed to a single CIE exposure (Figure 1A). Drinking sessions were suspended during the CIE exposure period and resumed following an 80‐h abstinence period. Mice that received the active (AAV‐shG9a) virus and control (AAV‐shCTRL) virus were separated into test groups: CIE‐alone or CIE + FSS groups for each vector condition. Of note, we did not include FSS‐alone or Air‐only groups given the lack of an effect of NAc G9a on baseline drinking from our previous study (Anderson et al., 2021) and a lack of FSS‐only effects in the CIE + FSS model (Anderson et al., 2016b; Haun et al., 2022; Lopez et al., 2020). CIE + FSS were performed as described above.

### Evaluating the Effects of Viral G9a Knockdown in the NAc on ethanol, water, and sucrose intake in two‐bottle choice assays

In a new group of mice, a NAc injection surgery was performed (AAV‐shCTRL vs. AAV‐shG9a); then, drinking sessions began 3 weeks later. Mice had access to 15% (v/v) ethanol in one bottle and water in a second bottle for 5 days per week for 2 h per day. Both ethanol intake and water intake were measured each day. In these same mice, we also measured sucrose consumption in a two‐bottle choice assay. We used 5% (w/v) sucrose diluted in water compared with water only and measured consumption for 2 h.

### Evaluating the effects of systemic administration of a G9a inhibitor

In eight new groups of mice, subjects were first given limited access alcohol drinking sessions for 4 weeks to establish stable baseline alcohol intake. The mice were then separated into four groups (CTL, FSS‐alone, CIE‐alone, CIE + FSS) and treated in the CIE + FSS drinking model as described above. In this study, mice received 4 alternating cycles of 1 week of CIE (or Air) exposure and then 5‐days of test volitional drinking sessions (Figure 3A). All mice were given injections (i.p.) of saline 30 min prior to the start of the 1‐h drinking sessions during the last week of baseline and each of the first three test drinking cycles. After the third CIE/Air exposure cycle, mice in the CTL, FSS‐alone, CIE‐alone, and CIE + FSS groups were injected with vehicle (0.1 mol/L sodium citrate buffer in saline) or UNC0642 (4 mg/kg, dissolved in vehicle) 30 min prior to each of the daily drinking sessions during the final 2 weeks of the experiment. The UNC0642 dosing was based on previous publications showing effective H3K9me2 reduction in vivo in mouse brain tissue (Wang et al., 2018).

### Statistics

For the CIE + FSS assays, average weekly alcohol intake (g/kg) served as the primary dependent variable. Two‐way ANOVAs, three‐way ANOVAs, or repeated measures (RM) three‐way ANOVAs were used to analyze behavioral data where appropriate. Newman–Keuls' post hoc tests were used following significant group differences. These CIE + FSS statistics were performed with Statistica (Tibco Software Inc.; Palo Alto, CA) and *p* < 0.05 was considered significant.

For the two‐bottle choice alcohol assay, average weekly alcohol intake (g/kg) or water intake (g/kg) served as the dependent variables. RM two‐way ANOVAs were used to analyze ethanol drinking, water drinking, and ethanol preference. For the two‐bottle choice sucrose assay, 5% (w/v) sucrose (g/kg) or water intake (g/kg) served as the dependent variables. T‐tests were used to analyze 5% (w/v) sucrose drinking, water drinking, and sucrose preference. These two‐bottle choice statistics were performed with Graphpad Prism, and *p* < 0.05 was considered statistically significant.

## RESULTS

### Viral‐mediated reduction of NAc G9a suppresses stress‐induced escalation of dependence‐related alcohol drinking

To determine whether reducing NAc G9a protein attenuates stress‐escalated alcohol drinking in the CIE + FSS model, we utilized a prevalidated adeno‐associated virus 2‐mediated short hairpin RNA (shRNA) interference approach to reduce endogenous G9a levels (AAV‐shG9a) as previously described (Anderson et al., 2019, 2021). As shown in the timeline (Figure 1A), 3 weeks following bilateral infusions of AAV‐shG9a or scrambled control, all mice were examined for baseline drinking (Figure 1B). Group (CIE, CIE + FSS) and Vector (AAV‐shCTRL, AAV‐shG9a) were the main between‐condition factors, while Time (baseline 1–4) was the repeated factor in this analysis. Similar to our previous results (Anderson et al., 2021), NAc shG9a had no significant effect on baseline drinking [no main effect of vector; *F*(1,45) = 0.55, *p* = 0.4643], suggesting that NAc G9a reduction is not sufficient to alter moderate, stable, baseline levels of alcohol drinking. The ANOVA also indicates a main effect of time [*F*(3,135) = 31.41, *p* < 0.0001] due to lower overall intake during baseline‐2 sessions compared with the other weeks and higher intake during baseline‐3 compared with the other weeks. Next, all mice were exposed to CIE for 1 week to increase blood–alcohol concentrations (BACs) overnight for 16 h. All CIE‐exposed mice had similar BACs showing that they were exposed to equivalent levels of alcohol and that an NAc G9a knockdown does not alter BACs (Table 1). An ANOVA with Group (CIE, CIE + FSS) and Vector (AAV‐shCTRL, AAV‐shG9a) as main factors did not indicate a significant effect of Group [*F*(1,43) = 0.27], Vector [*F*(1,43) = 2.11], or an interaction between these factors [*F*(1,43) = 0.001] (all *p* > 0.05). Next, all mice had 1‐h drinking sessions over the next 5 days, but half of the mice were exposed to FSS beginning at 4‐h prior to each drinking session. Group (CIE, CIE + FSS) and Vector (AAV‐shCTRL, AAV‐shG9a) were the main between‐condition factors, while Time (baseline, Test 1) was the repeated factor in this analysis. This ANOVA indicated significant main effects of Group [*F*(1,45) = 4.51, *p* < 0.05], Vector [*F*(1,45) = 4.85, *p* < 0.05], and Time [*F*(1,45) = 93.49, *p* < 0.0001]. The ANOVA also showed a significant interaction between Group and Time [*F*(1,45) = 10.88, *p* < 0.01] and a significant interaction between the three factors under analysis: Group, Vector, and Time [*F*(1,45) = 6.93, *p* < 0.025]. Post hoc comparisons based on the three‐way interaction term indicated that the control mice in the CIE + FSS group consumed more alcohol than the CIE‐only mice. In contrast, FSS did not further elevate alcohol consumption in the NAc shG9a group (Figure 1C). Finally, data expressed as percent change from baseline levels of intake were analyzed with Group (CIE, CIE + FSS) and Vector (AAV‐shCTRL and AAV‐shG9a) as main factors. This analysis indicated a main effect of Group [*F*(1,45) = 9.01, *p* < 0.01] and a significant interaction between Group and Vector [*F*(1,45) = 4.39, *p* < 0.05]. Post hoc comparisons showed that stress‐potentiated alcohol consumption was blocked in the NAc G9a knockdown mice (Figure 1D). In other words, only control mice (AAV‐shCTRL) showed significantly higher levels of alcohol consumption after FSS. These data are congruent with our previous findings demonstrating that the knockdown of NAc G9a reduces the ability of a pharmacological stressor to elevate alcohol drinking (Anderson et al., 2021).

**TABLE 1:** Blood ethanol concentrations during cycles of CIE in mice.

		AAV‐shCTRL	AAV‐shG9a
Figure 1	No stress	207.5 ± 5.9	195.0 ± 9.8
	FSS	203.2 ± 10.8	190.0 ± 8.5

	Cycle	noFSS/Vehicle	FSS/Vehicle	noFSS/UNC0642	FSS/UNC0642
Figure 2	1	174.0 ± 38.9	177.2 ± 38.3	164.1 ± 18.0	160.0 ± 25.0
	2	252.1 ± 26.7	247.5 ± 25.4	243.9 ± 22.5	246.8 ± 23.5
	3	324.3 ± 31.4	272.8 ± 17.8	236.1 ± 18.5	255.1 ± 20.8
	4	223.8 ± 17.0	228.6 ± 26.9	225.1 ± 17.5	173.8 ± 9.3

### Viral‐mediated reduction of NAc G9a levels does not alter alcohol, water, or sucrose drinking in two‐bottle choice assays

**FIGURE 2:**
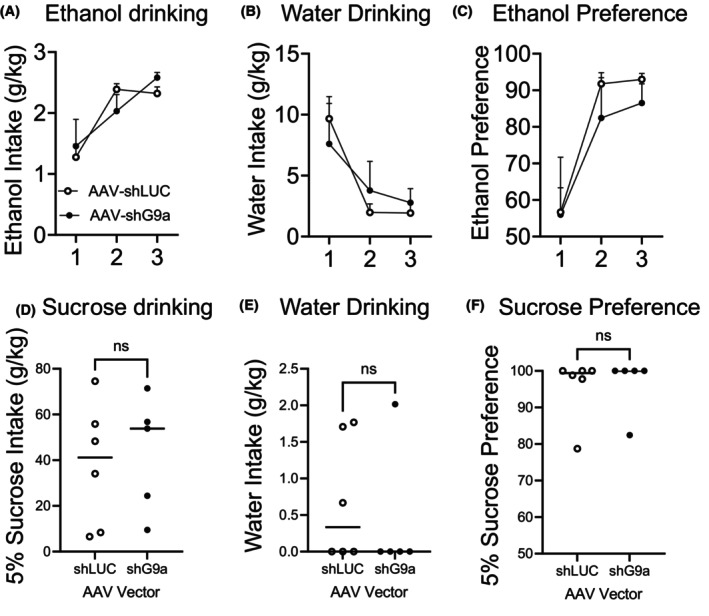
Accumbal G9a knockdown has no effect on baseline two‐bottle choice alcohol drinking, water drinking, or sucrose drinking. (A) Three weeks after a NAc AAV surgery, mice began 3 weeks of baseline testing for alcohol intake; (B): water intake; and (C) ethanol preference. (D) These same mice were also tested in a two‐bottle choice assay for 5% sucrose intake; (E) water intake; and (F) sucrose preference. Data are expressed as mean ± SEM. *N* = 5–6 male mice per group.

To test whether NAc G9a influences alcohol, water, or sucrose drinking and preference, we tested a new cohort of mice in a two‐bottle choice drinking paradigm. Following survival surgery to inject either AAV‐shCTRL or AAV‐shG9a bilaterally into the medial NAc, the mice were left in the home cage for at least 3 weeks for the virus to fully express before being allowed to drink ethanol or water. In contrast to heavy CIE or CIE + FSS ethanol drinking (Figure 1), we found no differences in moderate, baseline ethanol drinking between the groups, as both groups increased ethanol intake similarly over time (Figure 2A; vector: *F*_1,9_ = 0.01570, *p* = 0.2450; time: *F*_1.518,13.67_ = 17.53, *p* = 0.0003; interaction: *F*_2,18_ = 1.522, *p* = 0.9030). We also measured water intake and found that both groups decreased water intake over time similarly (Figure 2B; vector: *F*_1,9_ = 0.01190, *p* = 0.9155; time: *F*_1.149,10.34_ = 9.164, *p* = 0.0105; interaction: *F*_2,18_ = 0.7649, *p* = 0.4799). Also, we compared the water versus alcohol intake and found that both groups increased their preference for ethanol to a similar extent (Figure 2C; vector: *F*_1,9_ = 0.3691, *p* = 0.5585; time: *F*_1.269,11.42_ = 19.14, *p* = 0.0006; interaction: *F*_2,18_ = 0.2692, *p* = 0.7670). As an additional control, we next tested drinking of 5% (w/v) sucrose versus water in these same mice using a two‐bottle choice assay but found no differences in sucrose intake (Figure 2D, *t*(9) = 0.3288, *p* = 0.7498), water intake (Figure 2E, *t(*9) = 0.5419, *p* = 0.6010) or sucrose preference (Figure 2F, *t*(9) = 0.1224, *p* = 0.9053). Together, these results show that NAc G9a does not influence baseline alcohol, water, or sucrose drinking or preferences for alcohol or sucrose compared with water, and suggest that the NAc G9a‐dependent reductions in ethanol drinking following CIE + FSS are unlikely due to influences of G9a on ethanol consumption, ethanol preference, fluid intake, or general reward/tastant intake.

### Systemic treatment with a G9a inhibitor reduces both dependence‐induced and stress‐enhanced escalation of alcohol drinking

**FIGURE 3:**
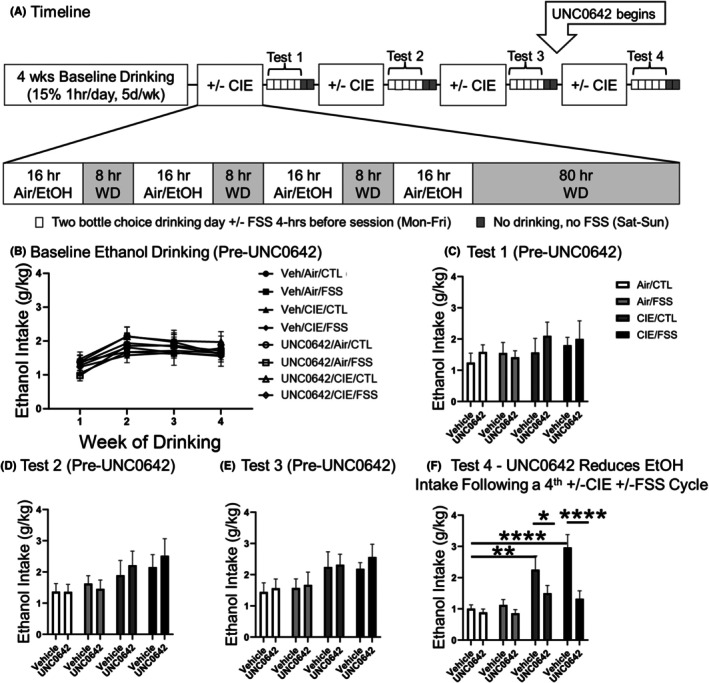
Systemic administration of the G9a inhibitor UNC0642 reduces both dependence‐induced escalation and stress + dependence‐induced escalation of ethanol drinking. (A) Timeline of experiment. UNC0642 versus vehicle injections began after the 3rd CIE + FSS session. (B) Average ethanol drinking at baseline. (C) Average ethanol drinking following the first CIE‐FSS, (D) Second CIE‐FSS cycle, and (E) Third CIE‐FSS cycle. (F) Average ethanol drinking following the fourth CIE‐FSS cycle. UNC0642 injections significantly reduced ethanol drinking in both the CIE‐alone and the CIE + FSS groups. Data are expressed as mean ± SEM. *p < 0.05; **p < 0.01; and ****p < 0.0001. *N* = 6–8 male mice per group.

We next tested whether systemic delivery of UNC0642, a potent, selective, brain‐permeable inhibitor of G9a (Liu et al., 2013), might also reduce stress‐escalated alcohol consumption in the CIE + FSS drinking model (Figure 3A). Mice were given limited access (1‐h/day) to 15% alcohol for 4 consecutive weeks to establish a stable baseline of alcohol intake. Mice were then divided into four groups: CTL (Air‐noFSS), FSS‐alone (Air‐FSS), CIE‐alone (CIE‐noFSS), and CIE + FSS. Following three alternating cycles of 1 week of CIE/Air and 1 week of test drinking, the groups were further separated before receiving systemic administration of UNC0642 (4 mg/kg; i.p.) or vehicle alone. We sorted the groups before UNC0642 administration to ensure similar baseline alcohol intake across the eight experimental groups. We used Group (CTL, FSS, CIE, CIE + FSS) and Drug (vehicle, UNC0642) as the main between‐condition factors versus Time (baseline 1–4) as the repeated factor in this factorial design. There was no statistical difference between the vehicle and UNC0642 groups within each +/‐CIE and +/‐FSS condition. The ANOVA indicates only a main effect of Time [*F*(3,135) = 22.63, *p* < 0.001] due to a lower overall alcohol intake during baseline‐1 compared with the rest of the baseline weeks (Figure 3B). Next, groups were exposed to +/−CIE and +/−FSS as indicated, but UNC0642 was not yet administered. To confirm that the groups had equivalent levels of ethanol drinking during the first 3 test cycles (i.e., before UNC0642 was administered), we analyzed these groups after each test session. Data were analyzed separately for each test cycle with Group (CTL, FSS, CIE, and CIE + FSS) and Drug (vehicle, UNC0642) as main factors. The ANOVA did not show a significant effect of these factors or significant interaction between them during Test 1 (Figure 3C). For Test 2, the ANOVA indicated a main effect of Group [*F*(3,45) = 2.87, *p* < 0.05] due to a higher intake in the CIE + FSS group compared with the CTL group (post hoc comparison: *p* = 0.055) (Figure 3D). For Test 3, there was a similar effect of Group [*F*(3,45) = 3.26, *p* < 0.05] again due to a higher intake in the CIE + FSS group compared with the CTL group (post hoc comparison: *p* = 0.07) (Figure 3E). Importantly, there was no effect of Drug since this was a “pseudo‐variable” at this point in the experiment (i.e., all groups received vehicle injections only). However, as shown in Figure 3F, injections of UNC0642 produced significant effects in Test 4 (Group [*F*(3,45) = 11.85, *p* < 0.001], Drug [*F*(1,45) = 15.15, *p* < 0.001], and a significant interaction between these factors [*F*(3,45) = 3.74, *p* < 0.05]). Post hoc comparisons showed a significantly elevated intake in vehicle‐treated CIE‐alone and CIE + FSS groups relative to the CTL and FSS‐alone groups that received vehicle. In addition, CIE + FSS mice likely consumed more alcohol than CIE‐alone mice, but it was only a statistical trend (post hoc comparison: *p* = 0.052). However, following repeated injections of UNC0642 (4 mg/kg; i.p.), drug‐treated mice drank significantly less alcohol in both CIE‐alone and CIE + FSS groups. In contrast, repeated UNC0642 injections did not alter alcohol intake in the CTL or FSS‐alone groups during this final week of testing (Figure 3F). Of note, CIE‐exposed mice had similar BACs (Table 1). The ANOVA with Group (CTL, FSS, CIE, CIE + FSS) and Drug (vehicle, UNC0642) as main between factors and Time (CIE cycle) indicated a main effect of time [*F*(3,63) = 14.82, *p* < 0.001] due to an overall lower BAC during the first CIE cycle compared with the following cycles and a lower BAC in the last cycle compared with Cycles 2 and 3. Nevertheless, no significant effects of Group, Drug, or interactions between these factors with Time were found, showing that these mice experienced similar levels of alcohol exposure before receiving treatment with UNC0642 on Tests 1–3. Also, these findings show that UNC0642 does not alter BACs following treatment (Table 1). Taken together, these results show that systemic administration of a G9a inhibitor selectively reduces CIE‐induced and FSS‐enhanced escalated alcohol drinking in the CIE + FSS drinking model.

## DISCUSSION

We found that both viral‐mediated reduction of NAc G9a levels and systemic inhibition of G9a activity reduce stress‐escalated alcohol drinking in male mice in a model of combined stress+dependence. Interestingly, we also found that systemic inhibition of G9a activity reduces alcohol drinking in male mice in the CIE model, but NAc G9a knockdown did not, similar to findings reported in our previous publication (Anderson et al., 2021). In control experiments, we found that NAc G9a does not influence alcohol preference, water consumption, sucrose consumption, or sucrose preference, suggesting that G9a's influence on stress‐potentiated alcohol drinking is not due to changes in fluid intake or reward sensitivity. Therefore, since stress and dependence are major factors for AUD, our preclinical findings here suggest that G9a inhibition could be a viable therapeutic strategy to reduce stress‐related heavy alcohol drinking in patients with AUD, though further testing is needed.

As mentioned above, we observed that viral‐mediated reduction of NAc G9a had no apparent impact on levels of alcohol drinking in CIE‐exposed C57BL/6J mice; however, systemic administration of UNC0642 did significantly reduce CIE‐escalated alcohol drinking. There are a number of possible explanations for this observation, including: (1) an insufficient reduction of NAc G9a levels using viral‐mediated knockdown compared with enzymatic inhibition, (2) effects of UNC0642 on additional targets, (3) the neuronal‐selective effects of AAV2‐shG9a, and/or (4) a role for G9a in brain or body regions beyond the NAc. First, viral vector expression issues could have caused an insufficient reduction of NAc G9a to effectively reduce CIE escalation of alcohol drinking. Our viral vector strategy did not infect all cells in the NAc due to viral efficiency and spread, so it is unlikely that we could reduce all neuronal NAc G9a. In contrast to this viral approach, the systemic administration of a G9a inhibitor may be able to reduce the activity of more NAc G9a proteins, thus effectively reducing CIE‐escalated drinking. One approach to test this hypothesis could be multiple injections of AAV vector in different NAc subregions to determine whether a larger knockdown reduces CIE‐escalated drinking. Second, another difference between our NAc G9a viral targeting and UNC0642 is their number of targets. While the NAc viral targeting strategy only reduces G9a due to the specificity of the shRNA base pairs, UNC0642 instead targets both G9a and G9a's binding partner called G9a‐like protein (GLP or EHMT1) due to their high homology and common binding site (Liu et al., 2013). Since GLP is also expressed in the NAc (Saunders et al., 2018; Savell et al., 2020), it is possible that inhibiting both GLP and G9a at the same time is necessary to reduce CIE‐escalated alcohol drinking. This could be tested in the future using an AAV‐shRNA knockdown approach to target GLP/EHMT1 alone or in combination with G9a knockdown. Additionally, a more selective drug that only inhibits G9a is possible, but the similarity in binding sites makes this less feasible. Third, another possibility is that our viral approach is more selectively targeting only neuronal G9a. There could also be an important role for non‐neuronal G9a in CIE‐escalated ethanol drinking, possibly located in astrocytes, microglia, or oligodendrocytes. Indeed, single‐cell nucleus databases from NAc or striatal tissues show that G9a and/or GLP is present in non‐neuronal cell types (Saunders et al., 2018, Savell et al., 2020). In fact, previous findings showing that NAc G9a is downregulated by alcohol were done with bulk NAc mRNA containing all these cell types (Anderson et al., 2021; Wolstenholme et al., 2011), so it is possible that some of those effects were driven by non‐neuronal changes in G9a. A new single‐cell analysis could illuminate the changes in G9a in both neuronal and non‐neuronal cells following chronic alcohol use, and non‐neuronal populations of G9a could also be targeted in future experiments. Lastly, another possibility is that G9a inhibitors are producing an effect somewhere else in the brain or the periphery, as several other studies have shown that G9a found in non‐NAc brain regions can play a role in alcohol‐related behaviors. For example, G9a is expressed in the amygdala and is altered by both morphine administration (Zhang et al., 2014) and alcohol administration, where it may alter anxiety behavior (Berkel et al., 2019). Interestingly, UNC0642 also reduces anxiety‐like behavior, as shown by several studies (Berkel et al., 2019; Wang et al., 2018) but NAc G9a also regulates anxiety behavior (Anderson et al., 2019; Anderson, Larson, et al., 2018), so it is possible that UNC0642‐induced anxiolysis is due to targeting accumbal G9a and not G9a in the amygdala. Alcohol also changes H3K9me2 levels in the PFC (De Clerck et al., 2024), basal forebrain (Crews et al., 2023; Crews & Vetreno, 2022), and hippocampus (Zou et al., 2022) suggesting other brain sites of action for UNC0642 as well. UNC0642 could also have peripheral effects, as a role for G9a in sensory neurons in the periphery has also been shown (Liang et al., 2016). Finally, non‐neuronal cells in the periphery express G9a, including breast and colon tissue, where G9a may play a role in some cancers (Kim et al., 2013; Shankar et al., 2013). Despite the potential roles for UNC0642 in other brain regions, there is still much evidence that UNC0642 acts in the NAc to reduce alcohol drinking behavior based on common behavioral effects of reducing stress‐potentiated drinking and anxiety.

The escalation of alcohol drinking caused by both dependence and stress may be due to a combined mechanism related to the effects of alcohol on the stress/KOR system. It is well established that CIE exposure increases stress reactivity and KOR signaling (Anderson et al., 2016a; Anderson & Becker, 2017; Becker et al., 2011; Liu & Weiss, 2002; Lopez et al., 2016). Since KOR signaling produces dysphoria during withdrawal and likely causes increased alcohol drinking by patients with AUD, the effects of NAc G9a and UNC0642 on KOR agonist‐induced stress‐escalated drinking seen in our previous study (Anderson et al., 2021), coupled with the effects of UNC0642 on CIE‐escalated drinking and CIE + FSS‐escalated alcohol drinking in this study, suggest a convergent mechanism. Of note, NAc‐specific KOR antagonists can reduce alcohol drinking in both alcohol self‐administering animals (Nealey et al., 2011) and in alcohol‐preferring rats (Uhari‐Vaananen et al., 2018). Though more studies are needed, it is possible that the G9a‐dependent reductions in alcohol drinking are due to NAc KOR signaling.

Since G9a is an epigenetic modifier, it is likely that G9a regulates stress‐potentiated alcohol drinking via epigenetic control of gene expression. G9a acts together with EHMT1 to dimethylate histone H3K9me2, and H3K9me2 is typically associated with gene repression (Anderson, Penrod, et al., 2018). Previous studies show that G9a manipulation in the NAc regulates many differentially expressed genes (Maze et al., 2010, 2014) that could potentially mediate stress‐potentiated alcohol drinking. For example, G9a regulates KOR and prodynorphin mRNA in medium spiny neurons in the NAc (Maze et al., 2014), suggesting a direct way for G9a to influence the dynorphin‐KOR pathway. However, numerous other targets could be playing a role, including corticotrophin‐releasing hormone receptor 1, protein kinase C alpha, or gamma‐aminobutyric acid type A receptor beta 1 (Maze et al., 2014), as these are genes that can alter stress‐ and/or alcohol‐related behavior (Anderson et al., 2016b; Anstee et al., 2013; Chen et al., 2015; Zorrilla et al., 2013). Since G9a regulates many genes simultaneously (Maze et al., 2014), it may not be possible to pinpoint a single gene responsible for these effects; instead, multiple targets may be acting together to regulate stress‐potentiated alcohol drinking. Future studies should examine the specific genes that are regulated by the interaction of stress, alcohol, and G9a to determine candidates to reduce stress‐potentiated alcohol drinking.

Although we focused on the therapeutic effects of G9a inhibition on alcohol in this study, G9a inhibition could be an effective strategy for SUDs as well. Both cocaine and opioids reduce NAc G9a expression (Maze et al., 2010; Sun et al., 2012) and we previously showed that NAc G9a knockdown decreases both cocaine‐taking behaviors and cocaine‐seeking behaviors (Anderson et al., 2019). Therefore, G9a inhibition might be therapeutic in humans that suffer from cocaine use disorder (CUD). In fact, given the high co‐occurrence between CUD and AUD (Althobaiti & Sari, 2016; Bierut et al., 2008; Taira et al., 1986), G9a inhibitors could be especially useful in treating this patient population.

Importantly, these studies used only male mice, so future studies should include female mice as well to test for potential sex differences, as there are well‐documented effects of sex on alcohol behaviors and AUD vulnerability (Agoglia et al., 2020; Ait‐Daoud et al., 2019; Finn et al., 2010; Peltier et al., 2019). The underlying reasons for these differences are poorly understood, but effective treatments for individuals suffering from AUD must be developed so that they are effective for patients with AUD regardless of sex or gender.

In sum, our findings suggest that NAc G9a is required for stress‐induced escalation of alcohol drinking. In addition, systemic administration of a G9a inhibitor, UNC0642, reduced both stress‐regulated and dependence‐escalated alcohol drinking in mice. Since G9a enzymatic inhibition can reduce alcohol drinking after the onset of dependence, this suggests that it might help to reduce alcohol consumption in patients that are dependent on alcohol. In contrast, neither NAc G9a reduction nor systemic G9a inhibition alters moderate, baseline levels of alcohol drinking, suggesting that G9a could potentially have clinical benefit for AUD patients without total abstinence. Since the stress system is dysregulated in chronic alcohol/substance abusers (Becker, 2012), these preclinical data suggest that chronic, systemic G9a inhibition could mitigate stress‐induced relapse and/or reduce heavy alcohol drinking in patients suffering from AUD, but more research is needed to test these possibilities.

## AUTHOR CONTRIBUTIONS

Each of the authors has contributed to this work in accordance with the criteria set in the guide to authors.

## CONFLICT OF INTEREST STATEMENT

ML and HB are consultants for NeuroEpigenix, LLC. CC and EA are co‐founders of NeuroEpigenix, LLC.

## FUNDING INFORMATION

This study was supported by the NIH (grant nos.: R41 AA029365, K01 DA046513, P50 AA10761, U24 AA029968, U01 AA014095, U01 AA020930, U29 AA020929, R01 DA032708, and R01 AA031007).

## Data Availability

The data that support the findings of this study are available from the corresponding author upon reasonable request.

## References

[acer70142-bib-0001] Agoglia, A.E. , Crofton, E.J. & Herman, M.A. (2020) Biological intersection of sex, age, and environment in the corticotropin releasing factor (CRF) system and alcohol. Neuropharmacology, 170, 108045. 32217364 10.1016/j.neuropharm.2020.108045 PMC7350391

[acer70142-bib-0002] Ait‐Daoud, N. , Blevins, D. , Khanna, S. , Sharma, S. , Holstege, C.P. & Amin, P. (2019) Women and addiction: an update. Medical Clinics of North America, 103, 699–711. 31078201 10.1016/j.mcna.2019.03.002

[acer70142-bib-0003] Althobaiti, Y.S. & Sari, Y. (2016) Alcohol interactions with psychostimulants: an overview of animal and human studies. Journal of Addiction Research & Therapy, 7, 281. 10.4172/2155-6105.1000281 PMC4966675 27478679

[acer70142-bib-0004] Anderson, E.M. , Larson, E.B. , Guzman, D. , Wissman, A.M. , Neve, R.L. , Nestler, E.J. et al. (2018) Overexpression of the histone dimethyltransferase G9a in nucleus accumbens shell increases cocaine self‐administration, stress‐induced reinstatement, and anxiety. The Journal of Neuroscience, 38, 803–813. 29217682 10.1523/JNEUROSCI.1657-17.2017 PMC5783964

[acer70142-bib-0005] Anderson, E.M. , Lopez, M.F. , Kastner, A. , Mulholland, P.J. , Becker, H.C. & Cowan, C.W. (2021) The histone methyltransferase G9a mediates stress‐regulated alcohol drinking. Addiction Biology, 27, e13060. 34013595 10.1111/adb.13060 PMC8602448

[acer70142-bib-0006] Anderson, E.M. , Penrod, R.D. , Barry, S.M. , Hughes, B.W. , Taniguchi, M. & Cowan, C.W. (2018) It's a complex issue: emerging connections between epigenetic regulators in drug addiction. The European Journal of Neuroscience, 50(3), 2477–2491. 30251397 10.1111/ejn.14170

[acer70142-bib-0007] Anderson, E.M. , Sun, H. , Guzman, D. , Taniguchi, M. , Cowan, C.W. , Maze, I. et al. (2019) Knockdown of the histone di‐methyltransferase G9a in nucleus accumbens shell decreases cocaine self‐administration, stress‐induced reinstatement, and anxiety. Neuropsychopharmacology, 44, 1370–1376. 30587852 10.1038/s41386-018-0305-4 PMC6785019

[acer70142-bib-0008] Anderson, E.M. & Taniguchi, M. (2022) Epigenetic effects of addictive drugs in the nucleus Accumbens. Frontiers in Molecular Neuroscience, 15, 828055. 35813068 10.3389/fnmol.2022.828055 PMC9260254

[acer70142-bib-0009] Anderson, E.M. , Tsvetkov, E. , Galante, A. , Devries, D. , Mccue, L.M. , Wood, D. et al. (2023) Epigenetic function during heroin self‐administration controls future relapse‐associated behavior in a cell type‐specific manner. Proceedings of the National Academy of Sciences of the United States of America, 120, e2210953120. 36745812 10.1073/pnas.2210953120 PMC9963300

[acer70142-bib-0010] Anderson, R.I. & Becker, H.C. (2017) Role of the dynorphin/kappa opioid receptor system in the motivational effects of ethanol. Alcoholism, Clinical and Experimental Research, 41, 1402–1418. 28425121 10.1111/acer.13406 PMC5522623

[acer70142-bib-0011] Anderson, R.I. , Lopez, M.F. & Becker, H.C. (2016a) Forced swim stress increases ethanol consumption in C57BL/6J mice with a history of chronic intermittent ethanol exposure. Psychopharmacology (Berlin), 233, 2035–2043. 26935824 10.1007/s00213-016-4257-2 PMC4864090

[acer70142-bib-0012] Anderson, R.I. , Lopez, M.F. & Becker, H.C. (2016b) Stress‐induced enhancement of ethanol intake in C57BL/6J mice with a history of chronic ethanol exposure: involvement of kappa opioid receptors. Frontiers in Cellular Neuroscience, 10, 45. 26941607 10.3389/fncel.2016.00045 PMC4763044

[acer70142-bib-0013] Anstee, Q.M. , Knapp, S. , Maguire, E.P. , Hosie, A.M. , Thomas, P. , Mortensen, M. et al. (2013) Mutations in the Gabrb1 gene promote alcohol consumption through increased tonic inhibition. Nature Communications, 4, 2816. 10.1038/ncomms3816 PMC3843143 24281383

[acer70142-bib-0014] Becker, H.C. (2012) Effects of alcohol dependence and withdrawal on stress responsiveness and alcohol consumption. Alcohol Research: Current Reviews, 34, 448–458. 23584111 10.35946/arcr.v34.4.09 PMC3860383

[acer70142-bib-0015] Becker, H.C. & Lopez, M.F. (2004) Increased ethanol drinking after repeated chronic ethanol exposure and withdrawal experience in C57BL/6 mice. Alcoholism, Clinical and Experimental Research, 28, 1829–1838. 15608599 10.1097/01.alc.0000149977.95306.3a

[acer70142-bib-0016] Becker, H.C. , Lopez, M.F. & Doremus‐Fitzwater, T.L. (2011) Effects of stress on alcohol drinking: a review of animal studies. Psychopharmacology, 218, 131–156. 21850445 10.1007/s00213-011-2443-9 PMC3247761

[acer70142-bib-0017] Berkel, T.D.M. , Zhang, H. , Teppen, T. , Sakharkar, A.J. & Pandey, S.C. (2019) Essential role of histone methyltransferase G9a in rapid tolerance to the anxiolytic effects of ethanol. International Journal of Neuropsychopharmacology, 22, 292–302. 30590608 10.1093/ijnp/pyy102 PMC6441132

[acer70142-bib-0018] Bierut, L.J. , Strickland, J.R. , Thompson, J.R. , Afful, S.E. & Cottler, L.B. (2008) Drug use and dependence in cocaine dependent subjects, community‐based individuals, and their siblings. Drug and Alcohol Dependence, 95, 14–22. 18243582 10.1016/j.drugalcdep.2007.11.023 PMC2384165

[acer70142-bib-0019] Chen, J. , Hutchison, K.E. , Calhoun, V.D. , Claus, E.D. , Turner, J.A. , Sui, J. et al. (2015) CREB‐BDNF pathway influences alcohol cue‐elicited activation in drinkers. Human Brain Mapping, 36, 3007–3019. 25939814 10.1002/hbm.22824 PMC4969622

[acer70142-bib-0020] Covington, H.E., 3rd , Maze, I. , Sun, H. , Bomze, H.M. , Demaio, K.D. , Wu, E.Y. et al. (2011) A role for repressive histone methylation in cocaine‐induced vulnerability to stress. Neuron, 71, 656–670. 21867882 10.1016/j.neuron.2011.06.007 PMC3163060

[acer70142-bib-0021] Crews, F.T. , Fisher, R.P. , Qin, L. & Vetreno, R.P. (2023) HMGB1 neuroimmune signaling and REST‐G9a gene repression contribute to ethanol‐induced reversible suppression of the cholinergic neuron phenotype. Molecular Psychiatry, 28, 5159–5172. 37402853 10.1038/s41380-023-02160-6 PMC10764639

[acer70142-bib-0022] Crews, F.T. & Vetreno, R.P. (2022) Cholinergic REST‐G9a gene repression through HMGB1‐TLR4 neuroimmune signaling regulates basal forebrain cholinergic neuron phenotype. Frontiers in Molecular Neuroscience, 15, 992627. 36072299 10.3389/fnmol.2022.992627 PMC9441808

[acer70142-bib-0055] Cruise, T.M. , Kotlo, K. , Malovic, E. & Pandey, S.C. (2023) Advances in DNA, histone, and RNA methylation mechanisms in the pathophysiology of alcohol use disorder. Advances in Drug and Alcohol Research, 3, 10871. Available from: doi: 10.3389/adar.2023.10871 38389820 PMC10880780

[acer70142-bib-0023] De Clerck, M. , Manguin, M. , Henkous, N. , D'almeida, M.N. , Beracochea, D. & Mons, N. (2024) Chronic alcohol‐induced long‐lasting working memory deficits are associated with altered histone H3K9 dimethylation in the prefrontal cortex. Frontiers in Behavioral Neuroscience, 18, 1354390. 38495426 10.3389/fnbeh.2024.1354390 PMC10941761

[acer70142-bib-0024] Finn, D.A. , Beckley, E.H. , Kaufman, K.R. & Ford, M.M. (2010) Manipulation of GABAergic steroids: sex differences in the effects on alcohol drinking‐ and withdrawal‐related behaviors. Hormones and Behavior, 57, 12–22. 19615369 10.1016/j.yhbeh.2009.07.002 PMC2813380

[acer70142-bib-0025] Grant, B.F. , Goldstein, R.B. , Saha, T.D. , Chou, S.P. , Jung, J. , Zhang, H. et al. (2015) Epidemiology of DSM‐5 alcohol use disorder: results from the National Epidemiologic Survey on alcohol and related conditions III. JAMA Psychiatry, 72, 757–766. 26039070 10.1001/jamapsychiatry.2015.0584 PMC5240584

[acer70142-bib-0026] Griffin, W.C., 3rd , Lopez, M.F. & Becker, H.C. (2009) Intensity and duration of chronic ethanol exposure is critical for subsequent escalation of voluntary ethanol drinking in mice. Alcoholism, Clinical and Experimental Research, 33, 1893–1900. 19673744 10.1111/j.1530-0277.2009.01027.x PMC2995298

[acer70142-bib-0027] Haun, H.L. , Lebonville, C.L. , Solomon, M.G. , Griffin, W.C. , Lopez, M.F. & Becker, H.C. (2022) Dynorphin/kappa opioid receptor activity within the extended amygdala contributes to stress‐enhanced alcohol drinking in mice. Biological Psychiatry, 91, 1019–1028. 35190188 10.1016/j.biopsych.2022.01.002 PMC9167153

[acer70142-bib-0028] Kim, Y. , Kim, Y.S. , Kim, D.E. , Lee, J.S. , Song, J.H. , Kim, H.G. et al. (2013) BIX‐01294 induces autophagy‐associated cell death via EHMT2/G9a dysfunction and intracellular reactive oxygen species production. Autophagy, 9, 2126–2139. 24322755 10.4161/auto.26308

[acer70142-bib-0029] Koob, G.F. & Volkow, N.D. (2016) Neurobiology of addiction: a neurocircuitry analysis. The Lancet Psychiatry, 3, 760–773. 27475769 10.1016/S2215-0366(16)00104-8 PMC6135092

[acer70142-bib-0030] Liang, L. , Zhao, J.Y. , Gu, X. , Wu, S. , Mo, K. , Xiong, M. et al. (2016) G9a inhibits CREB‐triggered expression of mu opioid receptor in primary sensory neurons following peripheral nerve injury. Molecular Pain, 12, 1744806916682242. 27927796 10.1177/1744806916682242 PMC5153028

[acer70142-bib-0031] Liu, F. , Barsyte‐Lovejoy, D. , Li, F. , Xiong, Y. , Korboukh, V. , Huang, X.P. et al. (2013) Discovery of an in vivo chemical probe of the lysine methyltransferases G9a and GLP. Journal of Medicinal Chemistry, 56, 8931–8942. 24102134 10.1021/jm401480r PMC3880643

[acer70142-bib-0032] Liu, X. & Weiss, F. (2002) Additive effect of stress and drug cues on reinstatement of ethanol seeking: exacerbation by history of dependence and role of concurrent activation of corticotropin‐releasing factor and opioid mechanisms. The Journal of Neuroscience, 22, 7856–7861. 12223538 10.1523/JNEUROSCI.22-18-07856.2002 PMC6758095

[acer70142-bib-0033] Lopez, M.F. , Anderson, R.I. & Becker, H.C. (2016) Effect of different stressors on voluntary ethanol intake in ethanol‐dependent and nondependent C57BL/6J mice. Alcohol, 51, 17–23. 26992696 10.1016/j.alcohol.2015.11.010 PMC4799834

[acer70142-bib-0034] Lopez, M.F. & Becker, H.C. (2005) Effect of pattern and number of chronic ethanol exposures on subsequent voluntary ethanol intake in C57BL/6J mice. Psychopharmacology, 181, 688–696. 16001125 10.1007/s00213-005-0026-3

[acer70142-bib-0035] Lopez, M.F. , Miles, M.F. , Williams, R.W. & Becker, H.C. (2017) Variable effects of chronic intermittent ethanol exposure on ethanol drinking in a genetically diverse mouse cohort. Alcohol, 58, 73–82. 27793543 10.1016/j.alcohol.2016.09.003 PMC5253308

[acer70142-bib-0036] Lopez, M.F. , Reasons, S.E. , Carper, B.A. , Nolen, T.L. , Williams, R.L. & Becker, H.C. (2020) Evaluation of the effect of doxasozin and zonisamide on voluntary ethanol intake in mice that experienced chronic intermittent ethanol exposure and stress. Alcohol, 89, 37–42. 32712186 10.1016/j.alcohol.2020.07.005 PMC7719616

[acer70142-bib-0037] Maze, I. , Chaudhury, D. , Dietz, D.M. , Von Schimmelmann, M. , Kennedy, P.J. , Lobo, M.K. et al. (2014) G9a influences neuronal subtype specification in striatum. Nature Neuroscience, 17, 533–539. 24584053 10.1038/nn.3670 PMC3972624

[acer70142-bib-0038] Maze, I. , Covington, H.E., 3rd , Dietz, D.M. , Laplant, Q. , Renthal, W. , Russo, S.J. et al. (2010) Essential role of the histone methyltransferase G9a in cocaine‐induced plasticity. Science, 327, 213–216. 20056891 10.1126/science.1179438 PMC2820240

[acer70142-bib-0039] Mokdad, A.H. , Marks, J.S. , Stroup, D.F. & Gerberding, J.L. (2004) Actual causes of death in the United States, 2000. JAMA, 291, 1238–1245. 15010446 10.1001/jama.291.10.1238

[acer70142-bib-0040] Nealey, K.A. , Smith, A.W. , Davis, S.M. , Smith, D.G. & Walker, B.M. (2011) Kappa‐opioid receptors are implicated in the increased potency of intra‐accumbens nalmefene in ethanol‐dependent rats. Neuropharmacology, 61, 35–42. 21338616 10.1016/j.neuropharm.2011.02.012 PMC3955175

[acer70142-bib-0056] Palmisano, M. & Pandey, S.C. (2017) Epigenetic mechanisms of alcoholism and stress‐related disorders. Alcohol, 60, 7–18. Available from:10.1016/j.alcohol.2017.01.001 28477725 PMC5464725

[acer70142-bib-0041] Peltier, M.R. , Verplaetse, T.L. , Mineur, Y.S. , Petrakis, I.L. , Cosgrove, K.P. , Picciotto, M.R. et al. (2019) Sex differences in stress‐related alcohol use. Neurobiology of Stress, 10, 100149. 30949562 10.1016/j.ynstr.2019.100149 PMC6430711

[acer70142-bib-0042] Sacks, J.J. , Gonzales, K.R. , Bouchery, E.E. , Tomedi, L.E. & Brewer, R.D. (2015) 2010 national and state costs of excessive alcohol consumption. American Journal of Preventive Medicine, 49, e73–e79. 26477807 10.1016/j.amepre.2015.05.031

[acer70142-bib-0043] Substance Abuse and Mental Health Services Administration . (2019) Key substance use and mental health indicators in the United States: Results from the 2018 National Survey on Drug Use and Health (HHS Publication No. PEP19‐5068, NSDUH Series H‐54). Rockville, MD: Center for Behavioral Health Statistics and Quality, Substance Abuse and Mental Health Services Administration. Available from: https://www.samhsa.gov/data/

[acer70142-bib-0044] Saunders, A. , Macosko, E.Z. , Wysoker, A. , Goldman, M. , Krienen, F.M. , de Rivera, H. et al. (2018) Molecular diversity and specializations among the cells of the adult mouse brain. Cell, 174, 1015–1030. 30096299 10.1016/j.cell.2018.07.028 PMC6447408

[acer70142-bib-0045] Savell, K.E. , Tuscher, J.J. , Zipperly, M.E. , Duke, C.G. , Phillips, R.A., 3rd , Bauman, A.J. et al. (2020) A dopamine‐induced gene expression signature regulates neuronal function and cocaine response. Science Advances, 6, eaba4221. 32637607 10.1126/sciadv.aba4221 PMC7314536

[acer70142-bib-0046] Shankar, S.R. , Bahirvani, A.G. , Rao, V.K. , Bharathy, N. , Ow, J.R. & Taneja, R. (2013) G9a, a multipotent regulator of gene expression. Epigenetics, 8, 16–22. 23257913 10.4161/epi.23331 PMC3549875

[acer70142-bib-0047] Sun, H. , Maze, I. , Dietz, D.M. , Scobie, K.N. , Kennedy, P.J. , Damez‐Werno, D. et al. (2012) Morphine epigenomically regulates behavior through alterations in histone H3 lysine 9 dimethylation in the nucleus accumbens. The Journal of Neuroscience, 32, 17454–17464. 23197736 10.1523/JNEUROSCI.1357-12.2012 PMC3516048

[acer70142-bib-0048] Taira, S. , Kakiuchi, T. , Minami, M. & Nariuchi, H. (1986) The regulatory role of sialic acids in the response of class II reactive T cell hybridomas to allogeneic B cells. The Journal of Immunology, 137, 2448–2454. 3531333

[acer70142-bib-0049] Uhari‐Vaananen, J. , Raasmaja, A. , Backstrom, P. , Oinio, V. , Carroll, F.I. , Airavaara, M. et al. (2018) The kappa‐opioid receptor antagonist JDTic decreases ethanol intake in alcohol‐preferring AA rats. Psychopharmacology (Berlin), 235, 1581–1591. 29492614 10.1007/s00213-018-4868-x

[acer70142-bib-0050] Wang, D.Y. , Kosowan, J. , Samsom, J. , Leung, L. , Zhang, K.L. , Li, Y.X. et al. (2018) Inhibition of the G9a/GLP histone methyltransferase complex modulates anxiety‐related behavior in mice. Acta Pharmacologica Sinica, 39, 866–874. 29417943 10.1038/aps.2017.190 PMC5943902

[acer70142-bib-0051] Wolstenholme, J.T. , Warner, J.A. , Capparuccini, M.I. , Archer, K.J. , Shelton, K.L. & Miles, M.F. (2011) Genomic analysis of individual differences in ethanol drinking: evidence for non‐genetic factors in C57BL/6 mice. PLoS One, 6, e21100. 21698166 10.1371/journal.pone.0021100 PMC3116881

[acer70142-bib-0052] Zhang, Z. , Tao, W. , Hou, Y.Y. , Wang, W. , Kenny, P.J. & Pan, Z.Z. (2014) MeCP2 repression of G9a in regulation of pain and morphine reward. The Journal of Neuroscience, 34, 9076–9087. 24990928 10.1523/JNEUROSCI.4194-13.2014 PMC4078085

[acer70142-bib-0053] Zorrilla, E.P. , Heilig, M. , de Wit, H. & Shaham, Y. (2013) Behavioral, biological, and chemical perspectives on targeting CRF(1) receptor antagonists to treat alcoholism. Drug and Alcohol Dependence, 128, 175–186. 23294766 10.1016/j.drugalcdep.2012.12.017 PMC3596012

[acer70142-bib-0054] Zou, J. , Walter, T.J. , Barnett, A. , Rohlman, A. , Crews, F.T. & Coleman, L.G., JR. (2022) Ethanol induces secretion of proinflammatory extracellular vesicles that inhibit adult hippocampal neurogenesis through G9a/GLP‐epigenetic signaling. Frontiers in Immunology, 13, 866073. 35634322 10.3389/fimmu.2022.866073 PMC9136051

